# Thirty-day suicidal thoughts and behaviours in the Spanish adult general population during the first wave of the Spain COVID-19 pandemic

**DOI:** 10.1017/S2045796021000093

**Published:** 2021-02-17

**Authors:** P. Mortier, G. Vilagut, M. Ferrer, I. Alayo, R. Bruffaerts, P. Cristóbal-Narváez, I. del Cura-González, J. Domènech-Abella, M. Felez-Nobrega, B. Olaya, J. I. Pijoan, E. Vieta, V. Pérez-Solà, R. C. Kessler, J. M. Haro, J. Alonso

**Affiliations:** 1 Health Services Research Unit, IMIM-Institut Hospital del Mar d'Investigacions Mèdiques, Barcelona, Spain; 2 CIBER Epidemiología y Salud Pública (CIBERESP), Madrid, Spain; 3 Universitat Autònoma de Barcelona (UAB), Barcelona, Spain; 4 Pompeu Fabra University, Barcelona, Spain; 5 Center for Public Health Psychiatry, Universitair Psychiatrisch Centrum, KU Leuven, Leuven, Belgium; 6 Parc Sanitari Sant Joan de Déu, Sant Boi de Llobregat, Barcelona, Spain; 7 CIBER de Salud Mental (CIBERSAM), Madrid, Spain; 8 Research Unit Primary Care Management, Madrid Health Service. (REDISSEC). Universidad Rey Juan Carlos, Madrid, Spain; 9 Clinical Epidemiology Unit, Hospital Universitario Cruces/ OSI EEC, Bilbao, Spain; 10 CIBER Epidemiología y Salud Pública (CIBERESP), Spain; 11 Hospital Clínic, University of Barcelona, IDIBAPS, (CIBERSAM), Barcelona, Spain; 12 Parc de Salut Mar PSMAR, Barcelona, Spain; 13 Department of Health Care Policy, Harvard Medical School, Boston, MA, USA; 14 CIBER Salud Mental (CIBERSAM), Madrid, Spain; 15 Universitat de Barcelona (UB), Barcelona, Spain; 16 Department of Experimental and Health Sciences, Pompeu Fabra University, Barcelona, Spain

**Keywords:** suicide, COVID-19, pandemic, epidemiology, risk factors, Spain

## Abstract

**Aims:**

To investigate the prevalence of suicidal thoughts and behaviours (STB; i.e. suicidal ideation, plans or attempts) in the Spanish adult general population during the first wave of the Spain coronavirus disease 2019 (COVID-19) pandemic (March−July, 2020), and to investigate the individual- and population-level impact of relevant distal and proximal STB risk factor domains.

**Methods:**

Cross-sectional study design using data from the baseline assessment of an observational cohort study (MIND/COVID project). A nationally representative sample of 3500 non-institutionalised Spanish adults (51.5% female; mean age = 49.6 [s.d. = 17.0]) was taken using dual-frame random digit dialing, stratified for age, sex and geographical area. Professional interviewers carried out computer-assisted telephone interviews (1–30 June 2020). Thirty-day STB was assessed using modified items from the Columbia Suicide Severity Rating Scale. Distal (i.e. pre-pandemic) risk factors included sociodemographic variables, number of physical health conditions and pre-pandemic lifetime mental disorders; proximal (i.e. pandemic) risk factors included current mental disorders and a range of adverse events-experiences related to the pandemic. Logistic regression was used to investigate individual-level associations (odds ratios [OR]) and population-level associations (population attributable risk proportions [PARP]) between risk factors and 30-day STB. All data were weighted using post-stratification survey weights.

**Results:**

Estimated prevalence of 30-day STB was 4.5% (1.8% active suicidal ideation; *n* = 5 [0.1%] suicide attempts). STB was 9.7% among the 34.3% of respondents with pre-pandemic lifetime mental disorders, and 1.8% among the 65.7% without any pre-pandemic lifetime mental disorder. Factors significantly associated with STB were pre-pandemic lifetime mental disorders (total PARP = 49.1%) and current mental disorders (total PARP = 58.4%), i.e. major depressive disorder (OR = 6.0; PARP = 39.2%), generalised anxiety disorder (OR = 5.6; PARP = 36.3%), post-traumatic stress disorder (OR = 4.6; PARP = 26.6%), panic attacks (OR = 6.7; PARP = 36.6%) and alcohol/substance use disorder (OR = 3.3; PARP = 5.9%). Pandemic-related adverse events-experiences associated with STB were lack of social support, interpersonal stress, stress about personal health and about the health of loved ones (PARPs 32.7–42.6%%), and having loved ones infected with COVID-19 (OR = 1.7; PARP = 18.8%). Up to 74.1% of STB is potentially attributable to the joint effects of mental disorders and adverse events−experiences related to the pandemic.

**Conclusions:**

STB at the end of the first wave of the Spain COVID-19 pandemic was high, and large proportions of STB are potentially attributable to mental disorders and adverse events−experiences related to the pandemic, including health-related stress, lack of social support and interpersonal stress. There is an urgent need to allocate resources to increase access to adequate mental healthcare, even in times of healthcare system overload.

**Study registration number:**

NCT04556565

## Introduction

The coronavirus disease 2019 (COVID-19) pandemic poses unprecedented challenges worldwide. In line with concerns about a potential increase in psychopathology related to the pandemic (Vindegaard and Benros, 2020), representative population-based studies (i.e. not using snowball sampling) from the Czech Republic, the UK and the US found high prevalence of suicidal thoughts and behaviours (STB) during the pandemic, i.e. suicidal ideation range 4.6–18% and suicidal behaviour range 0.1–4.9% (Bryan *et al*., 2020; Czeisler *et al*., 2020; Fitzpatrick *et al*., 2020; Gratz *et al*., 2020; Iob *et al*., 2020; O'Connor *et al*., 2020; Winkler *et al*., 2020; Ammerman *et al*., 2021). Mental health experts have therefore urged governments to invest resources in mental health and suicide prevention strategies (Gunnell *et al*., 2020; Moutier, 2020; Wasserman *et al*., 2020). To guide intervention planning, research is needed to provide reliable STB prevalence estimates and to quantify the population-level impact of relevant risk factors (Christensen *et al*., 2016). The latter can be achieved by calculating population attributable risk proportions (PARP; Krysinska and Martin 2009), i.e. estimates of potential reductions in STB when eliminating risk factors in the population. Despite their great value in prioritising prevention interventions (Christensen *et al*., 2016), no study to date provided PARP for STB during the COVID-19 pandemic.

Spain was hit particularly hard by the COVID-19 pandemic. The first COVID-19 case in Spain was confirmed on 31 January 2020. On 14 March, a state of alarm was declared, including national lockdown restrictions requiring citizens to stay at home except to purchase food and medicines, or to go to work. Schools and all non-essential shops and businesses were closed. As from 28 April, all activity in non-essential sectors was banned. Between the beginning of March and mid-April, daily new cases were >2000/day, peaking on 27 March (10 141 cases; WHO COVID-19 Dashboard). During the last two weeks of March, daily hospitalisations were >2000/day (RENAVE, 2020). Daily deaths peaked on 1 April (913 deaths) with 29 080 cumulative deaths by the end of May (WHO COVID-19 Dashboard). The healthcare system nearly collapsed during April−May due to lack of healthcare resources (RENAVE, 2020). By the time the situation stabilised in early July, Spain had the eighth highest number of cases (i.e. 249 659), and the fifth highest COVID death rate (i.e. 60.7/100 000) in the world (Roser *et al*., 2020).

In this report we investigate 30-day STB during the first wave of the COVID-19 pandemic (March−June 2020) in the Spanish adult general population. Spain traditionally has low rates of STB (12-month estimates 0.7–0.9%; Miret *et al*., 2014) and suicide (3.5–9.0/100 000; Alfonso-Sánchez *et al*., 2020), but this might have changed due to the particularly severe toll of COVID-19 in Spain in terms of number of cases and deaths, healthcare system overload and a prolonged period of national lockdown. No previous data exist on STB prevalence during the pandemic in Spain other than from an online survey (March 2020) based on non-representative snowball sampling (Sáiz *et al*., 2020). Representative estimates could contribute to the ongoing debate as to whether STB have increased during the pandemic. Here, we present data from a nationally representative sample assessed near the end of the first wave of the Spain COVID-19 pandemic (June 2020). By that time, daily new cases were <500/day, and restrictions were lifted to obtain a ‘new normality’, including social distancing, obligatory wearing of masks and limited capacity in shops, bars and restaurants.

Apart from STB prevalence, we also investigate a range of relevant STB risk factors for individual- and population-level impact. Distal risk factors (i.e. referring to the pre-pandemic period) include sociodemographic variables (Franklin *et al*., 2017; Carrasco-Barrios *et al*., 2020), physical health conditions (Franklin *et al*., 2017) and pre-pandemic mental disorders (Nock *et al*., 2015; Franklin *et al*., 2017; O'Connor *et al*., 2020). Proximal risk factors (i.e. referring to the pandemic period) are current mental disorders (Fitzpatrick *et al*., 2020; Iob *et al*., 2020); health-related factors, including COVID-19 infection (Iob *et al*., 2020; Winkler *et al*., 2020) and health-related stress (Winkler *et al*., 2020; Ammerman *et al*., 2021); financial factors (Fazel and Runeson, 2020), including COVID-19-related financial stress or job loss (Gratz *et al*., 2020; Winkler *et al*., 2020); and interpersonal factors, including interpersonal stress and lack of social support (O'Connor and Kirtley, 2018; Carrasco-Barrios *et al*., 2020).

## Methods

### Study design, population and sampling

This study is explanatory in purpose, and risk factors are therefore conceptualised as causal factors, i.e. factors that, when manipulated (e.g. through interventions), may change the outcome (Schooling and Jones, 2018). However, this is an exploratory observational study without experimental manipulation, using a cross-sectional study design. No temporality between the risk factors and the outcome can be established, and therefore, no causal inference can be made.

A baseline survey of a cohort of general population adults was conducted as part of the MIND/COVID project (MIND/COVID, 2020). The target population consisted of non-institutionalised Spanish adults (i.e. aged 18 years or older) without Spanish language barriers. Professional interviewers carried out computer-assisted telephone interviews (1–30 June 2020) in a sample drawn using dual-frame random digit dialing (*n* = 3500). Mobile numbers were generated through an automated system and landline numbers were selected from an internal database maintained by the survey company to ensure that all Spanish geographical areas were adequately represented. Up to seven calls at different times of the day and days of the week were attempted to each number. The distribution of the interviews was planned according to quotas in terms of age groups, sex and autonomous community (National Institute of Statistics in Spain, July 2019). A total of 138 656 numbers were sampled, with a final split of 71% mobile and 29% landline telephones; 45 002 numbers were non-eligible (i.e. non-existing numbers [ 43 120], numbers of enterprises [984], numbers of persons with Spanish language barriers [444], fax numbers [268] and numbers belonging to quota that were already completed [186]) and 72 428 had unknown eligibility (i.e. no contact was made after the seven attempted calls), resulting in a cooperation rate (i.e. the proportion of all cases interviewed of all eligible units ever contacted) of 16.5%.

Ethical approval was provided by the Parc de Salut Mar Clinical Research Ethics Committee (protocol 2020/9203/I) and by Parc Sanitari Sant Joan de Déu, Barcelona, Spain (PIC 86-20). Participants were fully informed about the objectives and procedures of the study prior to providing oral consent.

### Measures

A modified version of selected items from the Columbia Suicide Severity Rating Scale (Posner *et al*., 2011) assessed STB in the past 30 days, i.e. dichotomous items that assessed passive suicidal ideation (‘*wish you were dead or would go to sleep and never wake up*’), active suicidal ideation (‘*have thoughts of killing yourself*’), suicide plans (‘*think about how you might kill yourself* [*e.g. taking pills, shooting yourself*] *or work out a plan of how to kill yourself*’) and suicide attempt (‘*make a suicide attempt* [*i.e. purposefully hurt yourself with at least some intent to die*]). For analyses, three dichotomies were created: “any STB” (i.e. having any of the four STB outcomes), “passive suicidal ideation only” and “active suicidal ideation, plan or attempt”, with no STB as the reference level’.

Distal risk factor domains included in this study are socio-demographic variables, number of physical health conditions and type and number of pre-pandemic lifetime mental disorders. Proximal risk factor domains are type and number of current mental disorders as well as four risk factor domains considering adverse events-experiences related to the pandemic: personal health-related factors, factors related to the health of loved ones, financial factors and interpersonal factors.

Socio-demographic variables included age (i.e. 18–29/30–49/50–64/65 < years), sex, nationality (i.e. non-Spanish nationality or both Spanish and non-Spanish nationality, *v*. Spanish nationality only), marital status (i.e. single, divorced or legally separated, widowed and married), living with a partner, pre-pandemic level of income (i.e. <570/570–799/800–1049/1050–1299/1300–1549/1550–1799/1800–2199/2200–2699/2700–3599/3600–4499/4500–5999/6000 < euros), having children in care, having elderly people or people with a disability in care and work status (i.e. essential service worker, non-essential service worker, not working).

Physical health conditions were assessed using a 7-item checklist (Sangha *et al*., 2003) including respiratory diseases (not provoked by coronavirus), cardiovascular diseases, diabetes, cancer, chronic hepatic diseases, immunological diseases and ‘other’. A sum score was created and categorised into ‘none’, ‘exactly one’, ‘exactly two’ and ‘three or more’.

Pre-pandemic lifetime mental disorders were assessed using a checklist based on the Composite International Diagnostic Interview (CIDI; Kessler and Üstün 2004) that screens for depression, bipolar disorder, anxiety, panic attacks, alcohol and drug use problems and ‘other’ mental disorders. A sum score was created and categorised into ‘none’, ‘exactly one’ and ‘two or more’.

Current mental disorders were assessed using well-validated screener scales, i.e. two-week major depressive disorder (PHQ-8; cutoff score 10; Kroenke *et al*., 2009), two-week generalised anxiety disorder (GAD-7; cutoff score 10; Newman *et al*., 2002), 30-day panic attacks (adapted CIDI screening scale item; Kessler *et al*., 2013), 30-day post-traumatic stress disorder (4-item short form of the PCL-5; cutoff score 7; Zuromski *et al*., 2019) and 30-day alcohol and substance use disorders (CAGE-AID; cutoff score 2; Hinkin *et al*., 2001). A sum score was created and categorised into ‘none’, ‘exactly one’ and ‘two or more’.

Personal health-related factors included: a history of COVID-19 infection (positive test and/or medical diagnosis) and/or having been in isolation or quarantine related to COVID-19 (three items recoded into a dichotomy); number of close contacts (<1 m) when working outside of home (0–100); perceived inefficiency of available protective equipment at work (4-level Likert-type item ranging from ‘*sufficient*’ to ‘*completely insufficient*’); and personal health-related stress, i.e. a summary scale [0–4] of two 5-level Likert-type items (ranging from ‘*none*’ to ‘*very severe*’) adapted from the Peri Life Events Scale (PLES; Dohrenwend *et al*., 1978) assessing stress related to personal health and to potential COVID-19 infection.

Factors related to the health of loved ones included: having loved ones infected with COVID-19 (including type of loved one infected and severity of infection of most affected loved one); and stress related to loved ones' health, i.e. a summary scale [0–4] of two 5-level Likert-type adapted PLES items (ranging from ‘*none*’ to ‘*very severe*’) assessing stress related to the health of loved ones and to loved ones potentially getting infected with COVID-19 (Dohrenwend *et al*., 1978).

Financial factors included: a significant loss of income and/or [temporary] unemployment due to COVID-19 (two items recoded into a dichotomy); and financial stress, i.e. a summary scale [0–4] of two 5-level Likert-type adapted PLES items (ranging from ‘*none*’ to ‘*very severe*’) assessing financial stress and stress related to loss of job or income due to COVID-19 (Dohrenwend *et al*., 1978).

Interpersonal factors included: interpersonal stress, i.e. a summary scale [0–4] of four 5-level Likert-type adapted PLES items (ranging from ‘*none*’ to ‘*very severe*’) assessing stress related to romantic relationships, family relationships, other problems of loved ones and getting along with people at work (Dohrenwend *et al*., 1978); and lack of social support using the reverse scaled [0–4] Oslo Social Support Scale (Dalgard, 1996).

### Analysis

All analyses were conducted using SAS version 9.4 (SAS Institute Inc, 2013). Post-stratification weights were used to match the sample to the distribution of the Spain adult general population according to age groups, sex and geographic region. Item-level missing data were minimal (median 0.09% [IQR 0.03–0.25%]; see also online Supplementary Table 1) and addressed using single multivariable imputation by chained equations (van Buuren, 2018).

STB prevalence was estimated for the entire sample and stratified by distal risk factors and current mental disorders. Logistic regression was used to estimate individual-level associations (odds ratios [OR] with 95% CI) of the risk factors with the three STB outcomes. To deal with data sparseness, penalised maximum likelihood estimation (Firth-type estimation) was used (Allison, 2012). Population-level associations, i.e. population attributable risk proportions (PARP % [s.e.]; Krysinska and Martin 2009) were calculated using simulation methods based on the logistic regression equations. PARP is the proportion of the cumulative predicted value of an outcome explained statistically by specific predictor variables. If the odds ratios from the logistic regression equations represent causal effects of the risk factors under study, PARP can be interpreted as the expected proportional reduction in STB prevalence if STB risk factors were eradicated from the population.

Since causal relationships between the included risk factors are largely unknown, we refrained from constructing fully adjusted multivariable models to avoid the risk of overadjustment bias (Schisterman *et al*., 2009). A first series of analyses considered the distal risk factor domains. We estimated bivariable individual-level associations of socio-demographic variables and number of physical health conditions with each of the STB outcomes. Next, we estimated individual- and population-level associations of pre-pandemic lifetime mental disorders with STB using separate models for each type of disorder and for number of disorders, each time adjusting for sociodemographic variables and number of physical health conditions (i.e. the remaining distal risk factors). Unadjusted (bivariable) models are shown in the Supplement. We also calculated a total PARP representing the joint effects of type and number of pre-pandemic lifetime mental disorders using a model including seven dummy variables indicating the seven types of disorders plus one dummy variable indicating having two or more disorders (see Nock *et al*., 2015 for a detailed discussion of type-number models), again adjusting for the remaining distal risk factors.

A second series of analyses considered the proximal risk factors. We estimated individual- and population-level associations of proximal risk factors with STB using separate models for each proximal risk factor, each time adjusting for distal risk factors. Unadjusted (bivariable) models are shown in the Supplement. Next, we calculated total PARPs representing the joint effects of the risk factors belonging to each separate proximal risk factor domain, each time adjusting for all distal risk factors. Similar to the total PARP for pre-pandemic lifetime mental disorders, the total PARP for current mental disorders consisted of a type-number model including five dummy variables indicating the five types of disorders plus one dummy variable indicating having two or more disorders, adjusting for distal risk factors. We also calculated the total PARP representing the joint effects of all four risk factor domains considering pandemic adverse events−experiences, and the total PARP representing the joint effects of all five proximal risk factor domains, adjusting for distal risk factors.

## Results

### Thirty-day prevalence of STB

Estimated prevalence of any 30-day STB was 4.5% in the total sample, 9.7% among the 34.3% of respondents with pre-pandemic lifetime mental disorders and 1.8% among the 65.7% without any pre-pandemic lifetime mental disorder (Table 1; see online Supplementary Table 2 for unweighted and unimputed estimates). Around 40% of those reporting any 30-day STB reported active suicidal ideation, plans or attempts (1.8% in total); five respondents (0.1%) reported a suicide attempt. Sample characteristics are shown in Table 2 (columns 2 and 3; see online Supplementary Table 3 for unweighted and unimputed estimates). STB estimates stratified by distal risk factors and current mental disorders are shown in online Supplementary Table 4.

**Table 1.** Prevalence of 30-day STB in the Spanish adult general population during the first wave of the Spain COVID-19 pandemic (*n* = 3500)

	Full sample (*n* = 3500)			Among those without any pre-pandemic lifetime mental disorder (*n* = 2274)			Among those with any pre-pandemic lifetime mental disorder (*n* = 1226)		
	*n*^a^	%^a^	s.e.^a^	*n*^a^	%^a^	s.e.^a^	*n*^a^	%^a^	s.e.^a^
No STB	3340	95.5	0.4	2233	98.2	0.3	1107	90.3	0.9
Any STB	160	4.5	0.4	41	1.8	0.3	119	9.7	0.9
Passive suicidal ideation only	96	2.7	0.3	27	1.1	0.2	69	5.7	0.7
Active suicidal ideation, plan or attempt	64	1.8	0.2	14	0.7	0.2	50	4.0	0.6
- Active suicidal ideation only	20	0.6	0.1	3	0.1	0.1	17	1.5	0.4
- Suicide plan, no attempt	39	1.1	0.2	8	0.4	0.1	31	2.4	0.4
- Suicide attempt	5	0.1	0.1	3	0.1	0.1	2	0.1	0.1

Abbreviations: s.e. = standard error; STB = STB.

a Number of observations (*n*) are unweighted; proportions (%, s.e.) are weighted.

**Table 2.** Associations of distal risk factors (sociodemographic variables and number of physical health conditions) with 30-day STB (*n* = 3500)

			Any STB (*n* = 160)	Passive ideation only (*n* = 96)	Active suicidal ideation, plan or attempt (*n* = 64)
	*n*^a^	% (s.e.) or Med (s.e.) (IQR)^a^	OR (95% CI)^b^	OR (95% CI)^b^	OR (95% CI)^b^
**Sociodemographic variables**					
Age^c^					
65 years or more	622	23.7 (0.8)	0.7 (0.5–1.2)	1.2 (0.6–2.2)	0.3 (0.2–0.7)*
50–64 years	1127	25.8 (0.7)	0.7 (0.4–1.0)	0.9 (0.5–1.6)	0.5 (0.2–0.9)*
30–49 years	1305	36.0 (0.8)	0.5 (0.3–0.8)*	0.6 (0.3–1.1)	0.5 (0.2–0.9)*
18–29 years	446	14.6 (0.6)	(ref)	(ref)	(ref)
Being female	1962	51.5 (0.9)	1.8 (1.3–2.4)*	2.5 (1.6–3.9)*	1.1 (0.7–1.8)
Having non-Spanish nationality or both (vs. Spanish only)	331	9.4 (0.5)	1.1 (0.7–1.9)	1.3 (0.7–2.5)	0.9 (0.4–2.2)
Marital status					
Single	1180	35.3 (0.8)	1.4 (1.0–2.0)	1.0 (0.6–1.6)	2.2 (1.3–3.9)*
Divorced or legally separated	319	8.3 (0.5)	2.2 (1.3–3.6)*	1.8 (0.9–3.5)	3.0 (1.4–6.6)*
Widowed	195	6.7 (0.5)	1.8 (1.0–3.3)*	2.2 (1.1–4.2)*	1.2 (0.4–3.9)
Married	1806	49.7 (0.9)	(ref)	(ref)	(ref)
Living with a partner	2272	63.1 (0.8)	0.5 (0.4–0.7)*	0.7 (0.5–1.1)	0.3 (0.2–0.5)*
Having children in care	1195	31.1 (0.8)	0.7 (0.5–1.0)	0.6 (0.4–1.0)	0.9 (0.5–1.5)
Having elderly persons or persons with a disability in care	489	14.4 (0.6)	1.5 (1.0–2.2)	1.9 (1.2–3.0)*	1.0 (0.5–2.0)
Pre-pandemic level of income (scaled 1–12)		5.5 (0.1) (3.1–7.7)	0.9 (0.8–0.9)*	0.9 (0.8–0.9)*	0.9 (0.8–1.0)*
Work status					
Working, essential service worker	937	24.7 (0.7)	0.4 (0.2–0.6)*	0.4 (0.3–0.8)*	0.3 (0.1–0.7)*
Working, no essential service worker	769	20.9 (0.7)	0.4 (0.3–0.7)*	0.4 (0.2–0.7)*	0.5 (0.2–1.0)*
Not working	1794	54.5 (0.9)	(ref)	(ref)	(ref)
**Number of physical health conditions**					
Three or more	89	2.7 (0.3)	4.3 (2.3–8.2)*	3.9 (1.7–9.0)*	5.3 (2.2–12.7)*
Exactly two	295	8.4 (0.5)	3.1 (2.0–4.8)*	4.0 (2.4–6.8)*	1.8 (0.8–4.1)
Exactly one	997	28.6 (0.8)	1.3 (0.9–1.9)	1.2 (0.7–2.0)	1.5 (0.9–2.7)
None	2119	60.3 (0.9)	(ref)	(ref)	(ref)

Abbreviations: OR  =  odds ratio; CI = confidence interval; IQR = interquartile range; Med = median; s.e. = standard error; STB = suicidal thoughts and behaviours.

a Number of observations (*n*) are unweighted; proportions (%, s.e.) and medians (Med, IQR) are weighted.

b Bivariable models were used, i.e. a separate logistic regression model was created for each risk factor.

c Mean age = 49.6 years [standard deviation = 17.0] (weighted estimate).

* Indicate statistically significant results (*α* = 0.05).

### Associations of distal risk factors with STB

Bivariable associations of socio-demographic variables and number of physical health conditions with STB are shown in Table 2. Risk factors consistently associated with all three STB outcomes were pre-pandemic level of income (OR = 0.9), having work (ORs = 0.3–0.5) and having three or more physical health conditions (ORs = 3.9–5.3). Unique associations with active suicidal ideation, plan or attempt included being aged 30 or more (ORs = 0.3–0.5), being single (OR = 2.2), being divorced or legally separated (OR = 3.0) and living with a partner (OR = 0.3). Unique associations with passive suicidal ideation included being female (OR = 2.5), being widowed (OR = 2.2), having elderly persons or persons with a disability in care (OR = 1.9) and having exactly two physical health conditions (OR = 4.0).

Adjusted associations of pre-pandemic lifetime mental disorders with STB are shown in Table 3 (see online Supplementary Table 5 for unadjusted analyses). Almost all pre-pandemic disorders were significantly associated with all three STB outcomes (ORs = 2.7–21.0; PARPs = 2.8–48.7%), including comorbidity of disorders (ORs = 6.2–10.9; PARPs = 31.2–56.1%). Associations were generally stronger with active suicidal ideation, plan or attempt than with passive ideation only. Individual-level impact was particularly high for bipolar disorder (ORs = 9.3–21.0) while population-level impact was highest for depression and anxiety (PARPs = 33.9–48.7%). About 49.1% of any STB is potentially attributable to the joint effects of all pre-pandemic lifetime mental disorders.

**Table 3.** Associations of distal risk factors (pre-pandemic lifetime mental disorders) with 30-day STB (adjusted analyses; *n* = 3500)

			Any STB (*n* = 160)		Passive ideation only (*n* = 96)		Active suicidal ideation, plan or attempt (*n* = 64)	
	*n*^a^	% (s.e.)^a^	OR (95% CI)^b^	PARP % (s.e.)^b^	OR (95% CI)^b^	PARP % (s.e.)^b^	OR (95% CI)^b^	PARP % (s.e.)^b^
**Pre-pandemic lifetime mental disorders**								
Depression	490	13.6 (0.6)	5.4 (3.8–7.6)*	38.1 (4.6)*	4.7 (3.0–7.2)*	34.0 (6.1)*	6.7 (4.0–11.2)*	44.5 (7.1)*
Bipolar disorder	55	1.6 (0.2)	14.3 (7.8–26.3)*	11.3 (2.5)*	9.3 (4.2–20.8)*	7.6 (2.8)*	21.0 (9.7–45.5)*	16.7 (4.6)*
Panic attacks	199	5.5 (0.4)	5.6 (3.7–8.6)*	18.7 (3.4)*	4.7 (2.7–8.1)*	14.0 (4.2)*	7.5 (4.2–13.2)*	25.8 (6.1)*
Anxiety	1052	29.3 (0.8)	3.2 (2.3–4.4)*	39.7 (6.0)*	2.7 (1.8–4.1)*	33.9 (7.9)*	3.9 (2.4–6.6)*	48.7 (9.4)*
Alcohol use problems	37	1.0 (0.2)	5.1 (2.1–12.4)*	3.5 (1.5)*	4.5 (1.4–14.5)*	2.8 (1.7)*	6.2 (2.0–19.0)*	5.2 (2.9)*
Drug use problems (illicit drugs and/or medication)	50	1.6 (0.2)	5.5 (2.7–11.3)*	5.2 (1.7)*	6.8 (2.7–17.1)*	5.2 (2.2)*	4.4 (1.7–11.4)*	5.4 (3.0)
Other	30	0.8 (0.2)	3.0 (1.1–8.7)*	1.7 (1.1)	2.7 (0.7–10.9)	1.3 (1.3)	3.8 (1.0–14.1)*	2.7 (1.8)
Number of disorders								
Two or more	488	13.5 (0.6)	8.0 (5.4–12.1)*	41.5 (4.5)*	6.2 (3.7–10.5)*	31.2 (5.6)*	10.9 (6.0–19.7)*	56.1 (7.3)*
Exactly one	738	20.9 (0.7)	2.8 (1.8–4.3)*	15.8 (4.0)*	3.6 (2.2–6.0)*	24.0 (5.7)*	1.5 (0.7–3.3)	4.1 (5.2)
None	2274	65.7 (0.8)	(ref)	(ref)	(ref)	(ref)	(ref)	(ref)
**Total PARP**^c^				49.1 (5.4)*		48.3 (6.9)*		55.0 (8.5)*

Abbreviations: OR  =  odds ratio; CI = confidence interval; PARP = Population Attributable Risk Proportion; s.e. = standard error; STB = suicidal thoughts and behaviours.

a Number of observations (*n*) are unweighted; proportions (%, s.e.) are weighted.

b Adjusted models were used, i.e. a separate logistic regression model was created for each type of pre-pandemic lifetime mental disorder and for the number of pre-pandemic lifetime mental disorders, each time adjusting for sociodemographic variables and for number of physical health conditions.

c Total PARP considers the joint effects of type and number of pre-pandemic lifetime mental disorders, i.e. a model including seven dummy variables indicating the seven types of disorders plus one dummy variable indicating having two or more disorders, adjusting for sociodemographic variables and for number of physical health conditions.

* Indicate statistically significant results (*α* = 0.05); for PARP, statistical significance is based on the percentile bootstrap confidence interval.

### Associations of proximal risk factors with STB

Adjusted associations of proximal risk factors with 30-day STB are presented in Table 4 (see online Supplementary Table 6 for unadjusted analyses). Almost all current mental disorders were significantly associated with all three STB outcomes (ORs = 3.3–11.7; PARPs = 5.9–58.2%), including comorbidity of disorders (ORs = 9.4–36.5; PARPs = 40.7–68.7%). Considering personal health-related factors, we found that the perceived inefficiency of protective equipment at work was consistently associated with STB (ORs = 1.7–2.0; PARPs = 6.5–6.6%), while personal health-related stress was uniquely associated with active suicidal ideation, plan or attempt (OR = 1.7; PARP = 52.6%). Factors related to the health of loved ones were uniquely associated with passive suicidal ideation (ORs = 1.2–1.6; PARPs = 20.0–31.3%). Detailed analyses (online Supplementary Table 7) show that having a partner, child or parent infected (OR = 4.2 [95%CI 1.8–10.0]), or having a loved one with severe COVID-19 symptoms (OR = 3.1 [95%CI 1.5–6.4]) were also associated with active suicidal ideation, plan or attempt. No significant associations were found with financial factors. Interpersonal stress was consistently associated with STB (ORs = 1.4–1.9; PARPs = 30.7–50.0%) while lack of social support was uniquely associated with active suicidal ideation, plan or attempt (OR = 2.6; PARP = 68.3%).

**Table 4.** Associations of proximal risk factors with 30-day STB (adjusted analyses; *n* = 3500)

			Any STB (*n* = 160)		Passive ideation only (*n* = 96)		Active suicidal ideation, plan or attempt (*n* = 64)	
	*n*^a^	% (s.e.) or Med (s.e.) (IQR)^a^	OR (95% CI)^b^	PARP % (s.e.)^b^	OR (95% CI)^b^	PARP % (s.e.)^b^	OR (95% CI)^b^	PARP % (s.e.)^b^
**Positive screens for current mental disorders**								
Major depressive disorder	407	11.2 (0.5)	6.0 (4.1–8.8)*	39.2 (5.0)*	3.8 (2.4–6.1)*	27.4 (6.7)*	11.7 (6.5–20.8)*	58.2 (7.4)*
Generalised anxiety disorder	395	10.9 (0.5)	5.6 (3.8–8.1)*	36.3 (4.8)*	3.7 (2.3–5.9)*	25.4 (6.6)*	10.5 (5.9–18.7)*	54.0 (7.6)*
Post-traumatic stress disorder	342	9.5 (0.5)	4.6 (3.1–6.8)*	26.6 (4.3)*	4.2 (2.6–6.8)*	25.7 (5.5)*	4.8 (2.7–8.5)*	28.1 (7.2)*
Panic attacks	357	9.8 (0.5)	6.7 (4.5–10.0)*	36.6 (4.6)*	5.2 (3.2–8.5)*	29.9 (6.0)*	8.9 (4.9–16.2)*	47.3 (7.8)*
Alcohol or substance use disorder	94	2.8 (0.3)	3.3 (1.7–6.6)*	5.9 (2.6)*	1.3 (0.5–3.8)	1.8 (2.3)	5.0 (2.1–11.5)*	11.3 (5.0)*
Number of positive screens								
Two or more	426	11.8 (0.6)	15.6 (9.7–25.0)*	51.5 (4.3)*	9.4 (5.4–16.4)*	40.7 (5.8)*	36.5 (16.1–82.5)*	68.7 (6.2)*
Exactly one	418	11.8 (0.6)	7.1 (4.3–11.8)*	18.8 (3.7)*	5.7 (3.2–10.1)*	21.1 (5.1)*	10.3 (4.1–25.8)*	14.9 (4.8)*
None	2656	76.4 (0.7)	(ref)	(ref)	(ref)	(ref)	(ref)	(ref)
**Personal Health**								
History of COVID-19 infection or isolation/quarantine for COVID-19	600	16.5 (0.6)	0.8 (0.5–1.4)	−2.9 (2.7)	0.7 (0.4–1.4)	−3.6 (3.5)	1.0 (0.5–2.0)	0.9 (4.1)
Number of close contacts (<1 m) when working outside of home		0.0 (0.2) (0.0–0.0)	1.0 (1.0–1.0)	1.1 (1.1)	1.0 (1.0–1.0)	2.0 (1.5)	1.0 (1.0–1.0)	0.6 (2.8)
Perceived inefficiency of protective equipment at work (scaled 0–4)		0.0 (0.1) (0.0–0.0)	1.9 (1.4–2.4)*	6.5 (2.0)*	1.7 (1.3–2.4)*	6.6 (2.5)*	2.0 (1.4–2.9)*	6.4 (3.3)
Stress about personal health (scaled 0–4)		0.7 (0.0) (0.0–1.6)	1.4 (1.2–1.6)*	32.9 (8.7)*	1.2 (1.0–1.5)	16.0 (12.3)	1.7 (1.4–2.2)*	52.6 (11.9)*
**Health of loved ones**								
Having loved ones infected with COVID-19	1606	45.0 (0.9)	1.7 (1.2–2.3)*	18.8 (6.6)*	1.6 (1.1–2.5)*	20.0 (8.7)*	1.6 (0.9–2.7)	16.0 (11.0)
Stress about health loved ones (scaled 0–4)		1.7 (0.0) (0.8–2.6)	1.2 (1.1–1.4)*	32.7 (11.2)*	1.2 (1.0–1.5)*	31.3 (15.0)*	1.2 (1.0–1.5)	30.5 (18.1)
**Financial factors**								
Significant income loss or (temporarily) unemployed due to COVID-19	1445	39.8 (0.8)	1.1 (0.7–1.5)	1.9 (6.9)	1.2 (0.8–1.9)	6.7 (8.9)	0.9 (0.5–1.6)	−3.1 (12.1)
Stress about financial situation (scaled 0–4)		0.9 (0.0) (0.0–2.1)	1.1 (1.0–1.3)	14.9 (10.0)	1.1 (0.9–1.3)	10.9 (12.8)	1.2 (1.0–1.5)	22.0 (16.7)
**Interpersonal factors**								
Interpersonal stress (scaled 0–4)		0.6 (0.0) (0.0–1.4)	1.6 (1.3–1.9)*	38.9 (6.7)*	1.4 (1.2–1.8)*	30.7 (9.5)*	1.9 (1.4–2.4)*	50.0 (9.7)*
Lack of social support (scaled 0–4)		0.8 (0.0) (0.4–1.3)	1.7 (1.4–2.1)*	42.6 (9.2)*	1.3 (0.9–1.7)	14.4 (15.5)	2.6 (1.9–3.6)*	68.3 (10.0)*

Abbreviations: OR  =  odds ratio; CI = confidence interval; IQR = interquartile range; Med = median; PARP = Population Attributable Risk Proportion; s.e. = standard error; STB = suicidal thoughts and behaviours.

a Number of observations (*n*) are unweighted; proportions (%, s.e.) and medians (Med, IQR) are weighted.

b Adjusted models were used, i.e. a separate logistic regression model was created for each proximal risk factor, each time adjusting for distal risk factors.

* Indicate statistically significant results (*α* = 0.05); for PARP, statistical significance is based on the percentile bootstrap confidence interval.

Table 5 shows that large proportions of any 30-day STB are potentially attributable to interpersonal factors (total PARP = 62.4%), followed by current mental disorders (total PARP = 58.4%), factors related to the health of loved ones (total PARP = 42.2%) and to personal health (total PARP = 34.2%). Up to 69.7% of any STB is potentially attributable to the joint effects of pandemic adverse events-experiences, while up to 74.1% of STB is potentially attributable to the joint effects of all five proximal risk factor domains. Except for the health of loved ones domain, all risk factor domains were more strongly associated with active suicidal ideation, plan or attempt, than with passive suicidal ideation.

**Table 5.** Population-level associations of proximal risk factor domains with 30-day STB (*n* = 3500)

	Any STB (*n* = 160)	Passive ideation only (*n* = 96)	Active suicidal ideation, plan or attempt (*n* = 64)
	PARP % (s.e.)^a^	PARP % (s.e.)^a^	PARP % (s.e.)^a^
**Positive screens for current mental disorders**^b^	58.4 (3.9)*	45.6 (5.3)*	73.1 (5.0)*
**Adverse events-experiences related to the pandemic**			
Personal health	34.2 (8.6)*	18.1 (12.2)	54.4 (11.4)*
Health of loved ones	42.2 (11.3)*	41.6 (14.6)*	38.6 (20.1)
Financial factors	14.2 (10.4)	13.7 (13.8)	18.1 (18.0)
Interpersonal factors	62.4 (8.1)*	37.9 (14.8)*	81.4 (7.0)*
**Total all adverse events-experiences**^c^	69.7 (8.5)*	55.6 (14.2)*	83.0 (10.5)*
**Total all proximal risk factor domains**^d^	74.1 (7.7)*	56.4 (14.3)*	86.4 (8.5)*

Abbreviations: PARP = Population Attributable Risk Proportion; s.e. = standard error; STB = suicidal thoughts and behaviours.

a Adjusted models were used, i.e. a separate logistic regression model was created to estimate the joint effects of all STB risk factors belonging to the proximal risk factor domain under consideration, each time adjusting for distal risk factors.

b PARP considers the joint effects of type and number of current mental disorders, i.e. one model including five dummy variables indicating the five types of disorders plus one dummy variable indicating having two or more disorders, adjusting for distal risk factors.

c Total PARP considers the joint effects of all adverse events-experiences related to the pandemic, adjusting for distal risk factors. PARPs of separate adverse events-experiences domains do not add up to this total PARP because of the multifactorial aetiology of STB, i.e. one given risk factor can be part of multiple risk factor combinations and causal pathways leading to STB.

d Total PARP considers the joint effects of all proximal STB risk factors, adjusting for distal risk factors. PARPs of separate proximal risk factor domains do not add up to this total PARP because of the multifactorial aetiology of STB, i.e. one given risk factor can be part of multiple risk factor combinations and causal pathways leading to STB.

* Indicate statistically significant results (*α* = 0.05) based on the percentile bootstrap confidence interval.

## Discussion

Using data from a representative sample of general adults, 30-day prevalence of STB during the first wave of the Spain COVID-19 pandemic was estimated at 4.5%. Population-level impact analysis of risk factors showed that approximately three quarters of STB is potentially attributable to mental disorders and adverse events−experiences related to the pandemic, including health-related stress, lack of social support and interpersonal stress. Above all, these findings highlight the need for interventions that increase access to adequate mental healthcare.

Several limitations of this study need mentioning. First, the lack of a pre-pandemic reference point of STB precludes direct pre−post comparisons of STB prevalence. Second, the observational cross-sectional study design limits causal inference of the identified associations of risk factors with STB. Prospective studies are needed, which could also use a structural approach (e.g. direct acyclic graphs) to control for as much confounding as possible according to prior causal knowledge without introducing overadjustment bias. Third, poststratification weights were based on three sociodemographic variables only, and the survey cooperation rate was low (16.5%), which could have led to non-response bias. Scarce evidence from previous studies suggests that mental disorders are higher among survey non-respondents (Kessler *et al*., 1994), suggesting underestimation of STB in our study. Fourth, the assessment of mental disorders relied on self-report screener scales (current disorders) and a CIDI checklist (pre-pandemic lifetime disorders); it should be stressed that this assessment is inferior to face-to-face clinical assessments.

Thirty-day STB prevalence in this study (4.5%; about 1 in 22 adults) is substantially higher than reliable 12-month estimates in the pre-pandemic Spanish population (0.7–0.9%; Miret *et al*., 2014), even though lower than in representative population-based studies in other countries conducted during the COVID-19 pandemic: the Czech Republic (11.9%; Winkler *et al*., 2020), the UK (8.2–18.0%; Iob *et al*., 2020; O'Connor *et al*., 2020) and the US (4.6–15%; Bryan *et al*., 2020; Czeisler *et al*., 2020; Fitzpatrick *et al*., 2020; Gratz *et al*., 2020; Ammerman *et al*., 2021). Pre-pandemic studies also documented higher 12-month STB prevalence in countries such as the US (4.3%; SAMHSA, 2018) and the UK (5.4%; APMS, 2014) *v*. Spain. Apart from methodological differences (e.g. different STB measures), higher degrees of collectivism in Spain are likely to explain the lower STB in Spain relative to other countries (Eskin *et al*., 2020).

Despite our study lacking a direct pre-pandemic reference point of STB prevalence, two observations suggest a potential increase of STB during the pandemic. First, although ~75% of all STB was found among the roughly one-third of the population with a pre-pandemic history of psychopathology, STB among respondents without pre-pandemic mental disorders was substantial, i.e. 1.8%. Second, we found that 74.1% of STB is potentially attributable to mental disorders and adverse events−experiences related to the pandemic. This estimate is in line with the PARP that can be indirectly calculated when comparing the pre-pandemic STB estimate from Miret *et al*. (2014), i.e. 0.7–0.9%, with our STB estimate of 4.5%, i.e. PARP = (4.5−[0.7–0.9])/4.5 = 80.0–84.4% (Fleiss, 1979). Since no data on suicides during the Spain COVID pandemic are currently available, it is impossible to investigate whether the potential increase in STB that our study suggests is reflected in increased suicide rates. Studies from other countries suggest that suicide rates either remained unchanged during the first wave of the pandemic (e.g. Greece, Vandoros *et al*., 2020; the US, Faust *et al*., 2021; Australia, Coroners Court of Victoria, 2020; China, Qi *et al*., 2020) or decreased (e.g. Norway, Qin and Mehlum 2020; the UK, Office for National Statistics, 2020; Germany, Radeloff *et al*., 2021; Peru, Calderon-Anyosa and Kaufman 2021). A recent study from Japan found evidence for the so-called honeymoon effect, i.e. a delayed increase in suicide rates after an initial drop (Tanaka and Okamoto, 2021). Taken together, this warns for the adverse effects of the pandemic on current and future population mental health and urges policy makers to implement effective prevention intervention strategies (Moutier, 2020).

The strong associations of mental disorders and interpersonal factors with pandemic STB that our study documented are in line with pre-pandemic studies: a meta-analysis on STB risk factors in the general European population found a pooled OR = 7.4 for affective disorders, OR = 4.3 for anxiety disorders, OR = 1.5–2.5 for substance use and OR = 2.6 for low social support. Our findings also confirm pandemic STB studies that found health-related worries a significant risk factor (e.g. OR = 1.43 with 30-day suicide risk; Winkler *et al*., 2020). We now show that both stress about personal health and the health of loved ones is associated with 30-day STB. In contrast to previous studies (Gratz *et al*., 2020; Iob *et al*., 2020; Winkler *et al*., 2020), however, no significant associations were found with having a COVID-19 diagnosis or with financial factors. Given the scarce evidence available to date, more research is needed on the relationship between adverse events−experiences related to the pandemic and STB.

Our findings support recently proposed prevention frameworks targeting adverse mental health during and after the COVID-19 pandemic (Gunnell *et al*., 2020; Moutier, 2020; Wasserman *et al*., 2020). Above all, the results urge policy-makers to increase access to adequate mental healthcare in Spain, even during this time of healthcare system overload. The fact that nearly one out of 20 Spanish adults screened positive for STB points to the need for multi-stage routine suicide risk screening programmes in healthcare systems (Bahraini *et al*., 2020), and for augmenting the availability of crisis response helplines, safety planning and evidence-based treatments for STB (Gunnell *et al*., 2020; Moutier, 2020; Wasserman *et al*., 2020). In addition, one out of four Spanish adults screened positive for current mental disorders and roughly 58% of STB is potentially attributable to these disorders. Our findings also suggest an important role of pre-pandemic mental disorders with roughly 49% of STB being potentially attributable to disorders with onset before the pandemic. Given the high population-level burden these findings indicate, innovative methods such as remote tele-mental health services (Wasserman *et al*., 2020) or E-health interventions (Torok *et al*., 2020) are needed to support and complement traditional mental healthcare, not only during the pandemic, but also beyond (Vieta *et al*., 2020).

Our study also highlights the need for interventions supporting interpersonal relationships in the population. In line with previous research on prosocial behaviour under stress (Barzilay *et al*., 2020), stress related to the health of loved ones was higher than personal health-related stress, and having loved ones infected with COVID-19 – but not a personal COVID-19 infection – was strongly associated with STB. Furthermore, we found that tackling interpersonal stress and lack of social support in the population could potentially reduce STB up to ~62%. Taken together, these findings support previous calls (Gunnell *et al*., 2020; Moutier, 2020; Wasserman *et al*., 2020) to increase community-level support for those living alone, to enable regular (digital) check-ins by relatives and friends, to provide access to support by those experiencing health-related stress, family problems or domestic violence, as well as to implement public health strategies to promote stress resilience and cohesion. In conclusion, our findings together with emerging evidence worldwide call for a strong coordinated response from policy-makers, health care professionals and the scientific community to overcome current and prevent future adverse mental health related to the COVID-19 pandemic.

## Data Availability

The de-identified participant data as well as the study protocol, statistical analysis plan and data dictionaries used for this study are available as from publication and upon reasonable request from the corresponding author (PM; pmortier@imim.es) as long as the main objective of the data-sharing request is replicating the analysis and findings as reported in this paper (without investigator support), after approval of a proposal and with a signed data access agreement.

## Supplementary material

For supplementary material accompanying this paper visit https://doi.org/10.1017/S2045796021000093. click here to view supplementary material
