# Supplementary material for: Thirty-day suicidal thoughts and behaviours in the Spanish adult general population during the first wave of the Spain COVID-19 pandemic
Source: Epidemiol Psychiatr Sci. 2021 Feb 17;30:e19. doi: 10.1017/S2045796021000093 (PMC7925988; doi:10.1017/S2045796021000093)
Supplement: Supplementary file 1 [file S2045796021000093sup001.docx]

**Supplementary Table 1. Missingness per study variable (n=3,500).**

| **Variable** | **Label** | **N missing** | **% missing** |
| --- | --- | --- | --- |
| A1 | age | 0 | 0.00 |
| A2 | gender | 0 | 0.00 |
| A51 | nationality - Spanish | 0 | 0.00 |
| A52 | nationality - other | 0 | 0.00 |
| A7 | living with a partner | 0 | 0.00 |
| A8 | marital status | 0 | 0.00 |
| B1 | a history of COVID-19 infection - ever tested | 0 | 0.00 |
| B2 | a history of COVID-19 infection - tested positive | 8 | 0.23 |
| B3 | a history of COVID-19 infection - medical diagnosis | 5 | 0.14 |
| B10 | having loved ones infected with COVID-19 | 50 | 1.43 |
| B111 | having loved ones infected with COVID-19 - type of loved one - partner | 2 | 0.06 |
| B112 | having loved ones infected with COVID-19 - type of loved one - child(ren) | 2 | 0.06 |
| B113 | having loved ones infected with COVID-19 - type of loved one - parent(s) | 2 | 0.06 |
| B114 | having loved ones infected with COVID-19 - type of loved one - other family members | 2 | 0.06 |
| B115 | having loved ones infected with COVID-19 - type of loved one - friend(s) | 2 | 0.06 |
| B116 | having loved ones infected with COVID-19 - type of loved one - other(s) | 2 | 0.06 |
| B12 | having loved ones infected with COVID-19 - severity of infection of most affected loved one | 15 | 0.43 |
| B13 | having been in isolation or quarantine related to COVID-19 | 2 | 0.06 |
| C11 | pre-pandemic lifetime mental disorders - depression | 2 | 0.06 |
| C12 | pre-pandemic lifetime mental disorders - bipolar disorder | 2 | 0.06 |
| C13 | pre-pandemic lifetime mental disorders - panic attacks | 2 | 0.06 |
| C14 | pre-pandemic lifetime mental disorders - anxiety | 2 | 0.06 |
| C15 | pre-pandemic lifetime mental disorders - alcohol use problems | 2 | 0.06 |
| C16 | pre-pandemic lifetime mental disorders - drug use problems (illicit drugs and/or medication) | 2 | 0.06 |
| C17 | pre-pandemic lifetime mental disorders - other | 2 | 0.06 |
| C18 | pre-pandemic lifetime mental disorders - none | 2 | 0.06 |
| C2_1_scale | PHQ-8 item 1 | 10 | 0.29 |
| C2_2_scale | PHQ-8 item 2 | 10 | 0.29 |
| C2_3_scale | PHQ-8 item 3 | 15 | 0.43 |
| C2_4_scale | PHQ-8 item 4 | 12 | 0.34 |
| C2_5_scale | PHQ-8 item 5 | 10 | 0.29 |
| C2_6_scale | PHQ-8 item 6 | 9 | 0.26 |
| C2_7_scale | PHQ-8 item 7 | 10 | 0.29 |
| C2_8_scale | PHQ-8 item 8 | 16 | 0.46 |
| C3_1_scale | GAD-7 item 1 | 4 | 0.11 |
| C3_2_scale | GAD-7 item 2 | 4 | 0.11 |
| C3_3_scale | GAD-7 item 3 | 7 | 0.20 |
| C3_4_scale | GAD-7 item 4 | 6 | 0.17 |
| C3_5_scale | GAD-7 item 5 | 4 | 0.11 |
| C3_6_scale | GAD-7 item 6 | 5 | 0.14 |
| C3_7_scale | GAD-7 item 7 | 14 | 0.40 |
| C4_1_scale | PCL-5 item 1 | 7 | 0.20 |
| C4_2_scale | PCL-5 item 2 | 12 | 0.34 |
| C4_3_scale | PCL-5 item 3 | 0 | 0.00 |
| C4_4_scale | PCL-5 item 4 | 1 | 0.03 |
| C5 | CIDI screening scale item panic attacks | 15 | 0.43 |
| C6 | CAGE-AID item 1 | 1 | 0.03 |
| C7 | CAGE-AID item 2 | 5 | 0.14 |
| C8 | CAGE-AID item 3 | 3 | 0.09 |
| C9 | CAGE-AID item 4 | 4 | 0.11 |
| C10 | 30-day suicidal ideation passive | 2 | 0.06 |
| C111 | 30-day suicidal ideation active | 1 | 0.03 |
| C131 | 30-day suicide plan | 1 | 0.03 |
| C151 | 30-day suicide attempt | 2 | 0.06 |
| E1 | [temporary] unemployment due to COVID-19 | 0 | 0.00 |
| E2 | current work status - current working situation | 13 | 0.37 |
| E9 | pre-pandemic level of income | 0 | 0.00 |
| E10 | a significant loss of income | 11 | 0.31 |
| E12 | number of close contacts (<1 meter) when working outside of home | 70 | 2.00 |
| E16 | current work status - essential worker | 35 | 1.00 |
| E26 | perceived inefficiency of available protective equipment at work | 0 | 0.00 |
| F4_1_scale | number of children in care aged 0-5 | 0 | 0.00 |
| F4_2_scale | number of children in care aged 6-12 | 0 | 0.00 |
| F4_3_scale | number of children in care aged 13-18 | 0 | 0.00 |
| F4_4_scale | number of children in care aged 18+ | 0 | 0.00 |
| F5 | having elderly people or people with a disability in care | 0 | 0.00 |
| G1_1_scale | Peri Life Events Scale item 1 | 7 | 0.20 |
| G1_2_scale | Peri Life Events Scale item 2 | 8 | 0.23 |
| G1_4_scale | Peri Life Events Scale item 4 | 46 | 1.31 |
| G1_6_scale | Peri Life Events Scale item 6 | 6 | 0.17 |
| G1_7_scale | Peri Life Events Scale item 7 | 8 | 0.23 |
| G1_8_scale | Peri Life Events Scale item 8 | 32 | 0.91 |
| G1_9_scale | Peri Life Events Scale item 9 | 1 | 0.03 |
| G1_10_scale | Peri Life Events Scale item 10 | 14 | 0.40 |
| G1_11_scale | Peri Life Events Scale item 11 | 5 | 0.14 |
| G1_12_scale | Peri Life Events Scale item 12 | 0 | 0.00 |
| H1 | Oslo Social Support Scale item 1 | 23 | 0.66 |
| H2 | Oslo Social Support Scale item 2 | 0 | 0.00 |
| H3 | Oslo Social Support Scale item 3 | 87 | 2.49 |
| H111 | physical health conditions - respiratory diseases (not provoked by coronavirus) | 3 | 0.09 |
| H112 | physical health conditions - cardiovascular diseases | 3 | 0.09 |
| H113 | physical health conditions - diabetes | 3 | 0.09 |
| H114 | physical health conditions - cancer | 3 | 0.09 |
| H115 | physical health conditions - chronic hepatic diseases | 3 | 0.09 |
| H116 | physical health conditions - immunological diseases | 3 | 0.09 |
| H117 | physical health conditions - other | 3 | 0.09 |
|  |  |  |  |
|  | **MEDIAN** | 3 | 0.09 |
|  | **MIN** | 0 | 0.00 |
|  | **Q1** | 1 | 0.03 |
|  | **Q3** | 9 | 0.25 |
|  | **MAX** | 87 | 2.49 |

**Supplementary Tables 2. Prevalence of thirty-day suicidal thoughts and behaviors in the Spanish adult general population during the first wave of the Spain COVID-19 pandemic (n = 3,500).**

**Supplementary Table 2A. Unweighted STB estimates using the unimputed dataset.**

|  | **full sample** | | | **among those without any pre-pandemic lifetime mental disorder** | | | **among those with any pre-pandemic lifetime mental disorder** | | |
| --- | --- | --- | --- | --- | --- | --- | --- | --- | --- |
|  | **n^a^** | **%^a^** | **SE^a^** | **n^a^** | **%^a^** | **SE^a^** | **n^a^** | **%^a^** | **SE^a^** |
| no STB | 3338 | 95.5 | 0.4 | 2231 | 98.2 | 0.3 | 1105 | 90.4 | 0.8 |
| any STB | 159 | 4.5 | 0.4 | 41 | 1.8 | 0.3 | 118 | 9.6 | 0.8 |
| passive suicidal ideation only | 95 | 2.7 | 0.3 | 27 | 1.2 | 0.2 | 68 | 5.6 | 0.7 |
| active suicidal ideation, plan or attempt | 64 | 1.8 | 0.2 | 14 | 0.6 | 0.2 | 50 | 4.1 | 0.6 |
| - active suicidal ideation only | 20 | 0.6 | 0.1 | 3 | 0.1 | 0.1 | 17 | 1.4 | 0.3 |
| - suicide plan, no attempt | 39 | 1.1 | 0.2 | 8 | 0.4 | 0.1 | 31 | 2.5 | 0.4 |
| - suicide attempt | 5 | 0.1 | 0.1 | 3 | 0.1 | 0.1 | 2 | 0.2 | 0.1 |

a. number of observations (n) are unweighted; proportions (%, SE) are unweighted.

Abbreviations: SE = standard error; STB = suicidal thoughts and behaviors.

**Supplementary Table 2B. Weighted STB estimates using unimputed dataset.**

|  | **full sample** | | | **among those without any pre-pandemic lifetime mental disorder** | | | **among those with any pre-pandemic lifetime mental disorder** | | |
| --- | --- | --- | --- | --- | --- | --- | --- | --- | --- |
|  | **n^a^** | **%^a^** | **SE^a^** | **n^a^** | **%^a^** | **SE^a^** | **n^a^** | **%^a^** | **SE^a^** |
| no STB | 3339 | 95.5 | 0.4 | 2254 | 98.2 | 0.3 | 1083 | 90.3 | 0.9 |
| any STB | 158 | 4.5 | 0.4 | 42 | 1.8 | 0.3 | 116 | 9.7 | 0.9 |
| passive suicidal ideation only | 94 | 2.7 | 0.3 | 26 | 1.1 | 0.2 | 67 | 5.6 | 0.7 |
| active suicidal ideation, plan or attempt | 64 | 1.8 | 0.2 | 15 | 0.7 | 0.2 | 48 | 4.0 | 0.6 |
| - active suicidal ideation only | 21 | 0.6 | 0.1 | 3 | 0.1 | 0.1 | 17 | 1.5 | 0.4 |
| - suicide plan, no attempt | 39 | 1.1 | 0.2 | 9 | 0.4 | 0.1 | 29 | 2.4 | 0.4 |
| - suicide attempt | 5 | 0.1 | 0.1 | 3 | 0.1 | 0.1 | 2 | 0.1 | 0.1 |

a. number of observations (n) are weighted; proportions (%, SE) are weighted.

Abbreviations: SE = standard error; STB = suicidal thoughts and behaviors.

**Supplementary Table 2C. Unweighted STB estimates using the imputed dataset.**

|  | **full sample** | | | **among those without any pre-pandemic lifetime mental disorder** | | | **among those with any pre-pandemic lifetime mental disorder** | | |
| --- | --- | --- | --- | --- | --- | --- | --- | --- | --- |
|  | **n^a^** | **%^a^** | **SE^a^** | **n^a^** | **%^a^** | **SE^a^** | **n^a^** | **%^a^** | **SE^a^** |
| no STB | 3340 | 95.4 | 0.4 | 2233 | 98.2 | 0.3 | 1107 | 90.3 | 0.8 |
| any STB | 160 | 4.6 | 0.4 | 41 | 1.8 | 0.3 | 119 | 9.7 | 0.8 |
| passive suicidal ideation only | 96 | 2.7 | 0.3 | 27 | 1.2 | 0.2 | 69 | 5.6 | 0.7 |
| active suicidal ideation, plan or attempt | 64 | 1.8 | 0.2 | 14 | 0.6 | 0.2 | 50 | 4.1 | 0.6 |
| - active suicidal ideation only | 20 | 0.6 | 0.1 | 3 | 0.1 | 0.1 | 17 | 1.4 | 0.3 |
| - suicide plan, no attempt | 39 | 1.1 | 0.2 | 8 | 0.4 | 0.1 | 31 | 2.5 | 0.4 |
| - suicide attempt | 5 | 0.1 | 0.1 | 3 | 0.1 | 0.1 | 2 | 0.2 | 0.1 |

a. number of observations (n) are unweighted; proportions (%, SE) are unweighted.

Abbreviations: SE = standard error; STB = suicidal thoughts and behaviors.

**Supplementary Table 2D. Weighted STB estimates using the imputed dataset.**

|  | **full sample** | | | **among those without any pre-pandemic lifetime mental disorder** | | | **among those with any pre-pandemic lifetime mental disorder** | | |
| --- | --- | --- | --- | --- | --- | --- | --- | --- | --- |
|  | **n^a^** | **%^a^** | **SE^a^** | **n^a^** | **%^a^** | **SE^a^** | **n^a^** | **%^a^** | **SE^a^** |
| no STB | 3342 | 95.5 | 0.4 | 2256 | 98.2 | 0.3 | 1085 | 90.3 | 0.9 |
| any STB | 159 | 4.5 | 0.4 | 42 | 1.8 | 0.3 | 117 | 9.7 | 0.9 |
| passive suicidal ideation only | 95 | 2.7 | 0.3 | 26 | 1.1 | 0.2 | 68 | 5.7 | 0.7 |
| active suicidal ideation, plan or attempt | 64 | 1.8 | 0.2 | 15 | 0.7 | 0.2 | 48 | 4.0 | 0.6 |
| - active suicidal ideation only | 21 | 0.6 | 0.1 | 3 | 0.1 | 0.1 | 17 | 1.5 | 0.4 |
| - suicide plan, no attempt | 39 | 1.1 | 0.2 | 9 | 0.4 | 0.1 | 29 | 2.4 | 0.4 |
| - suicide attempt | 5 | 0.1 | 0.1 | 3 | 0.1 | 0.1 | 2 | 0.1 | 0.1 |

a. number of observations (n) are weighted; proportions (%, SE) are weighted.

Abbreviations: SE = standard error; STB = suicidal thoughts and behaviors.

**Supplementary Table 3. Distribution of sample characteristics (i.e., sociodemographic variables).**

|  | **Unweighted estimates using unimputed dataset** | | **Weighted estimates using unimputed dataset** | | **Unweighted estimates using imputed dataset** | | **Weighted estimates using imputed dataset** | |
| --- | --- | --- | --- | --- | --- | --- | --- | --- |
|  | **n** | **% (SE) or Med (SE) (IQR)** | **n** | **% (SE) or Med (SE) (IQR)** | **n** | **% (SE) or Med (SE) (IQR)** | **n** | **% (SE) or Med (SE) (IQR)** |
| **Sociodemographic variables** |  |  |  |  |  |  |  |  |
| Age |  |  |  |  |  |  |  |  |
| 65 years or more | 622 | 17.8 (0.6) | 829 | 23.7 (0.8) | 622 | 17.8 (0.6) | 829 | 23.7 (0.8) |
| 50-64 years | 1127 | 32.2 (0.8) | 902 | 25.8 (0.7) | 1127 | 32.2 (0.8) | 902 | 25.8 (0.7) |
| 30-49 years | 1305 | 37.3 (0.8) | 1259 | 36.0 (0.8) | 1305 | 37.3 (0.8) | 1259 | 36.0 (0.8) |
| 18-29 years | 446 | 12.7 (0.6) | 510 | 14.6 (0.6) | 446 | 12.7 (0.6) | 510 | 14.6 (0.6) |
| Being female | 1962 | 56.1 (0.8) | 1802 | 51.5 (0.9) | 1962 | 56.1 (0.8) | 1802 | 51.5 (0.9) |
| Having non-Spanish nationality or both (vs. Spanish only) | 331 | 9.5 (0.5) | 330 | 9.4 (0.5) | 331 | 9.5 (0.5) | 330 | 9.4 (0.5) |
| Marital status |  |  |  |  |  |  |  |  |
| single | 1180 | 33.7 (0.8) | 1236 | 35.3 (0.8) | 1180 | 33.7 (0.8) | 1236 | 35.3 (0.8) |
| divorced or legally separated | 319 | 9.1 (0.5) | 289 | 8.3 (0.5) | 319 | 9.1 (0.5) | 289 | 8.3 (0.5) |
| widowed | 195 | 5.6 (0.4) | 235 | 6.7 (0.5) | 195 | 5.6 (0.4) | 235 | 6.7 (0.5) |
| married | 1806 | 51.6 (0.8) | 1740 | 49.7 (0.9) | 1806 | 51.6 (0.8) | 1740 | 49.7 (0.9) |
| Living with a partner | 2272 | 64.9 (0.8) | 2207 | 63.1 (0.8) | 2272 | 64.9 (0.8) | 2207 | 63.1 (0.8) |
| Having children in care | 1195 | 34.1 (0.8) | 1089 | 31.1 (0.8) | 1195 | 34.1 (0.8) | 1089 | 31.1 (0.8) |
| Having elderly persons or persons with a disability in care | 489 | 14.0 (0.6) | 505 | 14.4 (0.6) | 489 | 14.0 (0.6) | 505 | 14.4 (0.6) |
| Pre-pandemic level of income (scaled 1-12) |  | 5.5 (0.1) (3.1-7.6) |  | 5.5 (0.1) (3.1-7.6) |  | 5.5 (0.1) (3.1-7.7) |  | 5.5 (0.1) (3.1-7.7) |
| Work status |  |  |  |  |  |  |  |  |
| working, essential service worker | 921 | 26.7 (0.8) | 849 | 24.6 (0.7) | 937 | 26.8 (0.7) | 864 | 24.7 (0.7) |
| working, no essential service worker | 745 | 21.6 (0.7) | 706 | 20.4 (0.7) | 769 | 22.0 (0.7) | 730 | 20.9 (0.7) |
| not working | 1786 | 51.7 (0.9) | 1898 | 55.0 (0.9) | 1794 | 51.3 (0.8) | 1906 | 54.5 (0.9) |

Abbreviations: IQR = interquartile range; Med = median; SE = standard error.

**Supplementary Table 4. STB prevalence rates total, and stratified by sociodemographic variables, number of physical health conditions, and by pre-pandemic lifetime and current mental disorders.**

|  |  |  | **Any STB (n = 160))** | | **Passive ideation only (n = 96)** | | **Active suicidal ideation, plan or attempt (n = 64)** | |
| --- | --- | --- | --- | --- | --- | --- | --- | --- |
|  | **n^a^** | **% (SE)^a^** | **n^a^** | **% (SE)^a^** | **n^a^** | **% (SE)^a^** | **n^a^** | **% (SE)^a^** |
| **Total** |  |  |  | 4.5 (0.4) |  | 2.7 (0.3) |  | 1.8 (0.2) |
| **Sociodemographic factors** |  |  |  |  |  |  |  |  |
| Age |  |  |  |  |  |  |  |  |
| 65 years or more | 622 | 23.7 (0.8) | 30 | 4.9 (0.9) | 23 | 3.7 (0.8) | 7 | 1.2 (0.4) |
| 50-64 years | 1127 | 25.8 (0.7) | 52 | 4.4 (0.6) | 32 | 2.7 (0.5) | 20 | 1.7 (0.4) |
| 30-49 years | 1305 | 36.0 (0.8) | 49 | 3.6 (0.5) | 27 | 1.9 (0.4) | 22 | 1.7 (0.4) |
| 18-29 years | 446 | 14.6 (0.6) | 29 | 6.5 (1.2) | 14 | 3.0 (0.8) | 15 | 3.5 (0.9) |
| *Rao-Scott (R-S) ꭓ2 test or Fisher-Exact (F-E) test^b^* |  |  | *R-S ꭓ2 (3) =7.19, p=0.066* | | *R-S ꭓ2 (3) =6.13, p=0.105* | | *F-E p=0.072* | |
| Sex - female | 1962 | 51.5 (0.9) | 111 | 5.7 (0.5) | 73 | 3.8 (0.5) | 38 | 1.9 (0.3) |
| Sex - male | 1538 | 48.5 (0.9) | 49 | 3.3 (0.5) | 23 | 1.5 (0.3) | 26 | 1.8 (0.3) |
| *Rao-Scott (R-S) ꭓ2 test or Fisher-Exact (F-E) test^b^* |  |  | *R-S ꭓ2 (1) =10.17, p=0.001** | | *R-S ꭓ2 (1) =14.97, p< 0.001** | | *R-S ꭓ2 (1) =0.05, p=0.818* | |
| Nationality - non-Spanish nationality or both | 331 | 9.4 (0.5) | 19 | 5.0 (1.1) | 13 | 3.4 (0.9) | 6 | 1.6 (0.6) |
| Nationality - Spanish nationality only | 3169 | 90.6 (0.5) | 141 | 4.5 (0.4) | 83 | 2.6 (0.3) | 58 | 1.8 (0.2) |
| *Rao-Scott (R-S) ꭓ2 test or Fisher-Exact (F-E) test^b^* |  |  | *R-S ꭓ2 (1) =0.20, p=0.653* | | *R-S ꭓ2 (1) =0.74, p=0.391* | | *F-E p=1.000* | |
| Marital status |  |  |  |  |  |  |  |  |
| single | 1180 | 35.3 (0.8) | 58 | 4.9 (0.6) | 29 | 2.4 (0.4) | 29 | 2.5 (0.5) |
| divorced or legally separated | 1806 | 49.7 (0.9) | 65 | 3.6 (0.4) | 44 | 2.4 (0.4) | 21 | 1.1 (0.3) |
| widowed | 319 | 8.3 (0.5) | 26 | 7.4 (1.4) | 14 | 4.1 (1.1) | 12 | 3.3 (0.9) |
| married | 195 | 6.7 (0.5) | 11 | 6.2 (1.8) | 9 | 5.0 (1.6) | 2 | 1.2 (0.8) |
| *Rao-Scott (R-S) ꭓ2 test or Fisher-Exact (F-E) test^b^* |  |  | *R-S ꭓ2 (3) =10.17, p=0.017** | | *F-E p=0.077* | | *F-E p=0.003** | |
| Living with a partner - yes | 2272 | 63.1 (0.8) | 81 | 3.5 (0.4) | 57 | 2.4 (0.3) | 24 | 1.0 (0.2) |
| Living with a partner - no | 1228 | 36.9 (0.8) | 79 | 6.3 (0.7) | 39 | 3.2 (0.5) | 40 | 3.2 (0.5) |
| *Rao-Scott (R-S) ꭓ2 test or Fisher-Exact (F-E) test^b^* |  |  | *R-S ꭓ2 (1) =14.62, p< 0.001** | | *R-S ꭓ2 (1) =1.49, p=0.222* | | *R-S ꭓ2 (1) =20.21, p< 0.001** | |
| Having children in care - yes | 1195 | 31.1 (0.8) | 45 | 3.6 (0.5) | 25 | 1.9 (0.4) | 20 | 1.7 (0.4) |
| Having children in care - no | 2305 | 68.9 (0.8) | 115 | 4.9 (0.5) | 71 | 3.1 (0.4) | 44 | 1.9 (0.3) |
| *Rao-Scott (R-S) ꭓ2 test or Fisher-Exact (F-E) test^b^* |  |  | *R-S ꭓ2 (1) =3.25, p=0.071* | | *R-S ꭓ2 (1) =4.09, p=0.043** | | *R-S ꭓ2 (1) =0.15, p=0.699* | |
| Having elderly persons or persons with a disability in care - yes | 489 | 14.4 (0.6) | 32 | 6.1 (1.1) | 23 | 4.4 (0.9) | 9 | 1.7 (0.6) |
| Having elderly persons or persons with a disability in care - no | 3011 | 85.6 (0.6) | 128 | 4.3 (0.4) | 73 | 2.4 (0.3) | 55 | 1.8 (0.3) |
| *Rao-Scott (R-S) ꭓ2 test or Fisher-Exact (F-E) test^b^* |  |  | *R-S ꭓ2 (1) =3.14, p=0.076* | | *R-S ꭓ2 (1) =6.22, p=0.013** | | *F-E p=1.000* | |
| Work status |  |  |  |  |  |  |  |  |
| working, essential service worker | 1794 | 54.5 (0.9) | 117 | 6.2 (0.6) | 68 | 3.7 (0.5) | 49 | 2.5 (0.4) |
| working, no essential service worker | 769 | 20.9 (0.7) | 21 | 2.7 (0.6) | 12 | 1.4 (0.4) | 9 | 1.2 (0.4) |
| not working | 937 | 24.7 (0.7) | 22 | 2.4 (0.5) | 16 | 1.6 (0.4) | 6 | 0.7 (0.3) |
| *Rao-Scott (R-S) ꭓ2 test or Fisher-Exact (F-E) test^b^* |  |  | *R-S ꭓ2 (2) =27.59, p< 0.001** | | *R-S ꭓ2 (2) =16.15, p< 0.001** | | *F-E p< 0.001** | |
| **Number of physical health conditions** |  |  |  |  |  |  |  |  |
| three or more | 89 | 2.7 (0.3) | 12 | 13.0 (3.7) | 6 | 6.6 (2.7) | 6 | 6.4 (2.6) |
| exactly two | 295 | 8.4 (0.5) | 29 | 9.8 (1.8) | 23 | 7.6 (1.6) | 6 | 2.2 (0.9) |
| exactly one | 997 | 28.6 (0.8) | 43 | 4.5 (0.7) | 22 | 2.4 (0.5) | 21 | 2.1 (0.5) |
| none | 2119 | 60.3 (0.9) | 76 | 3.4 (0.4) | 45 | 2.0 (0.3) | 31 | 1.4 (0.3) |
| *Rao-Scott (R-S) ꭓ2 test or Fisher-Exact (F-E) test^b^* |  |  | *R-S ꭓ2 (3) =37.35, p< 0.001** | | *F-E p< 0.001** | | *F-E p=0.011** | |
| **Pre-pandemic lifetime mental disorders** |  |  |  |  |  |  |  |  |
| depression - yes | 490 | 13.6 (0.6) | 81 | 16.2 (1.7) | 44 | 9.1 (1.3) | 37 | 7.2 (1.2) |
| depression - no | 3010 | 86.4 (0.6) | 79 | 2.7 (0.3) | 52 | 1.7 (0.2) | 27 | 1.0 (0.2) |
| *Rao-Scott (R-S) ꭓ2 test or Fisher-Exact (F-E) test^b^* |  |  | *R-S ꭓ2 (1) =164.59, p< 0.001** | | *R-S ꭓ2 (1) =79.93, p< 0.001** | | *R-S ꭓ2 (1) =81.77, p< 0.001** | |
| bipolar disorder - yes | 55 | 1.6 (0.2) | 22 | 39.2 (6.7) | 8 | 16.3 (5.3) | 14 | 22.9 (5.6) |
| bipolar disorder - no | 3445 | 98.4 (0.2) | 138 | 4.0 (0.3) | 88 | 2.5 (0.3) | 50 | 1.5 (0.2) |
| *Rao-Scott (R-S) ꭓ2 test or Fisher-Exact (F-E) test^b^* |  |  | *R-S ꭓ2 (1) =147.97, p< 0.001** | | *F-E p< 0.001** | | *R-S ꭓ2 (1) =139.04, p< 0.001** | |
| panic attacks - yes | 199 | 5.5 (0.4) | 41 | 20.0 (2.9) | 19 | 9.3 (2.1) | 22 | 10.7 (2.2) |
| panic attacks - no | 3301 | 94.5 (0.4) | 119 | 3.6 (0.3) | 77 | 2.3 (0.3) | 42 | 1.3 (0.2) |
| *Rao-Scott (R-S) ꭓ2 test or Fisher-Exact (F-E) test^b^* |  |  | *R-S ꭓ2 (1) =110.17, p< 0.001** | | *R-S ꭓ2 (1) =33.19, p< 0.001** | | *R-S ꭓ2 (1) =86.82, p< 0.001** | |
| anxiety - yes | 1052 | 29.3 (0.8) | 99 | 9.3 (0.9) | 54 | 5.2 (0.7) | 45 | 4.2 (0.6) |
| anxiety - no | 2448 | 70.7 (0.8) | 61 | 2.5 (0.3) | 42 | 1.7 (0.3) | 19 | 0.8 (0.2) |
| *Rao-Scott (R-S) ꭓ2 test or Fisher-Exact (F-E) test^b^* |  |  | *R-S ꭓ2 (1) =72.56, p< 0.001** | | *R-S ꭓ2 (1) =31.20, p< 0.001** | | *R-S ꭓ2 (1) =40.80, p< 0.001** | |
| alcohol use disorder - yes | 37 | 1.0 (0.2) | 8 | 20.8 (6.7) | 4 | 9.5 (4.6) | 4 | 11.3 (5.4) |
| alcohol use disorder - no | 3463 | 99.0 (0.2) | 152 | 4.4 (0.4) | 92 | 2.6 (0.3) | 60 | 1.7 (0.2) |
| *Rao-Scott (R-S) ꭓ2 test or Fisher-Exact (F-E) test^b^* |  |  | *F-E p< 0.001** | | *F-E p=0.018** | | *F-E p=0.004** | |
| drug use disorder (illicit drugs and/or medication) - yes | 50 | 1.6 (0.2) | 11 | 21.4 (5.9) | 6 | 11.3 (4.5) | 5 | 10.1 (4.3) |
| drug use disorder (illicit drugs and/or medication) - no | 3450 | 98.4 (0.2) | 149 | 4.3 (0.4) | 90 | 2.6 (0.3) | 59 | 1.7 (0.2) |
| *Rao-Scott (R-S) ꭓ2 test or Fisher-Exact (F-E) test^b^* |  |  | *R-S ꭓ2 (1) =32.99, p< 0.001** | | *F-E p=0.002** | | *F-E p=0.002** | |
| other disorder - yes | 30 | 0.8 (0.2) | 5 | 14.6 (6.3) | 2 | 6.5 (4.4) | 3 | 8.2 (4.7) |
| other disorder - no | 3470 | 99.2 (0.2) | 155 | 4.4 (0.4) | 94 | 2.7 (0.3) | 61 | 1.8 (0.2) |
| *Rao-Scott (R-S) ꭓ2 test or Fisher-Exact (F-E) test^b^* |  |  | *F-E p=0.011** | | *F-E p=0.198* | | *F-E p=0.017** | |
| Number of pre-pandemic lifetime mental disorders |  |  |  |  |  |  |  |  |
| -two or more | 488 | 13.5 (0.6) | 81 | 16.3 (1.7) | 38 | 7.7 (1.2) | 43 | 8.6 (1.3) |
| -exactly one | 738 | 20.9 (0.7) | 38 | 5.5 (0.9) | 31 | 4.4 (0.8) | 7 | 1.1 (0.4) |
| -none | 2274 | 65.7 (0.8) | 41 | 1.8 (0.3) | 27 | 1.1 (0.2) | 14 | 0.7 (0.2) |
| *Rao-Scott (R-S) ꭓ2 test or Fisher-Exact (F-E) test^b^* |  |  | *R-S ꭓ2 (2) =178.12, p< 0.001** | | *R-S ꭓ2 (2) =69.60, p< 0.001** | | *F-E p< 0.001** | |
| **Positive screens for current mental disorders** |  |  |  |  |  |  |  |  |
| Major Depressive Disorder - yes | 407 | 11.2 (0.5) | 87 | 21.3 (2.1) | 43 | 10.2 (1.5) | 44 | 11.1 (1.6) |
| Major Depressive Disorder - no | 3093 | 88.8 (0.5) | 73 | 2.4 (0.3) | 53 | 1.8 (0.2) | 20 | 0.6 (0.1) |
| *Rao-Scott (R-S) ꭓ2 test or Fisher-Exact (F-E) test^b^* |  |  | *R-S ꭓ2 (1) =274.65, p< 0.001** | | *R-S ꭓ2 (1) =90.65, p< 0.001** | | *R-S ꭓ2 (1) =206.00, p< 0.001** | |
| Generalized Anxiety Disorder - yes | 395 | 10.9 (0.5) | 85 | 20.9 (2.1) | 41 | 10.0 (1.5) | 44 | 10.9 (1.6) |
| Generalized Anxiety Disorder - no | 3105 | 89.1 (0.5) | 75 | 2.5 (0.3) | 55 | 1.8 (0.2) | 20 | 0.7 (0.2) |
| *Rao-Scott (R-S) ꭓ2 test or Fisher-Exact (F-E) test^b^* |  |  | *R-S ꭓ2 (1) =251.25, p< 0.001** | | *R-S ꭓ2 (1) =83.50, p< 0.001** | | *R-S ꭓ2 (1) =178.38, p< 0.001** | |
| Post-Traumatic Stress Disorder - yes | 342 | 9.5 (0.5) | 66 | 18.6 (2.1) | 38 | 10.6 (1.7) | 28 | 8.0 (1.5) |
| Post-Traumatic Stress Disorder - no | 3158 | 90.5 (0.5) | 94 | 3.1 (0.3) | 58 | 1.9 (0.3) | 36 | 1.2 (0.2) |
| *Rao-Scott (R-S) ꭓ2 test or Fisher-Exact (F-E) test^b^* |  |  | *R-S ꭓ2 (1) =161.60, p< 0.001** | | *R-S ꭓ2 (1) =83.40, p< 0.001** | | *R-S ꭓ2 (1) =75.99, p< 0.001** | |
| Panic Attacks - yes | 357 | 9.8 (0.5) | 83 | 22.6 (2.3) | 43 | 11.5 (1.7) | 40 | 11.1 (1.7) |
| Panic Attacks - no | 3143 | 90.2 (0.5) | 77 | 2.6 (0.3) | 53 | 1.8 (0.2) | 24 | 0.8 (0.2) |
| *Rao-Scott (R-S) ꭓ2 test or Fisher-Exact (F-E) test^b^* |  |  | *R-S ꭓ2 (1) =272.42, p< 0.001** | | *R-S ꭓ2 (1) =107.87, p< 0.001** | | *R-S ꭓ2 (1) =170.31, p< 0.001** | |
| Alcohol or Substance Use Disorder - yes | 94 | 2.8 (0.3) | 19 | 19.1 (4.1) | 8 | 7.1 (2.5) | 11 | 12.0 (3.5) |
| Alcohol or Substance Use Disorder - no | 3406 | 97.2 (0.3) | 141 | 4.1 (0.4) | 88 | 2.6 (0.3) | 53 | 1.5 (0.2) |
| *Rao-Scott (R-S) ꭓ2 test or Fisher-Exact (F-E) test^b^* |  |  | *R-S ꭓ2 (1) =46.14, p< 0.001** | | *F-E p=0.004** | | *R-S ꭓ2 (1) =51.49, p< 0.001** | |
| Number of positive screens for current mental disorder |  |  |  |  |  |  |  |  |
| -two or more | 426 | 11.8 (0.6) | 96 | 22.2 (2.1) | 48 | 10.9 (1.5) | 48 | 11.3 (1.6) |
| -exactly one | 418 | 11.8 (0.6) | 33 | 8.6 (1.5) | 23 | 6.1 (1.3) | 10 | 2.5 (0.8) |
| -none | 2656 | 76.4 (0.7) | 31 | 1.2 (0.2) | 25 | 0.9 (0.2) | 6 | 0.2 (0.1) |
| *Rao-Scott (R-S) ꭓ2 test or Fisher-Exact (F-E) test^b^* |  |  | *R-S ꭓ2 (2) =355.03, p< 0.001** | | *R-S ꭓ2 (2) =143.72, p< 0.001** | | *F-E p< 0.001** | |

a. number of observations (n) are unweighted; proportions (%, SE) are weighted.

b. Fisher-Exact (F-E) test was used in case of cell counts < 10.

SE = standard error; STB = suicidal thoughts and behaviors.

* indicate statistically significant results (α = 0.05).

**Supplementary Table 5. Associations of distal risk factors (pre-pandemic lifetime mental disorders) with thirty-day STB (unadjusted analyses; n = 3,500).**

|  |  |  | **Any STB (n = 160)** | | **Passive ideation only (n = 96)** | | **Active suicidal ideation, plan or attempt (n = 64)** | |
| --- | --- | --- | --- | --- | --- | --- | --- | --- |
|  | **n^a^** | **% (SE)^a^** | **OR (95% CI)^b^** | **PARP % (SE)^b^** | **OR (95% CI)^b^** | **PARP % (SE)^b^** | **OR (95% CI)^b^** | **PARP % (SE)^b^** |
| **Pre-pandemic lifetime mental disorders** |  |  |  |  |  |  |  |  |
| depression | 490 | 13.6 (0.6) | 7.0 (5.0-9.7)* | 41.2 (4.6)* | 6.2 (4.1-9.3)* | 37.5 (6.0)* | 8.5 (5.1-13.9)* | 46.8 (7.0)* |
| bipolar disorder | 55 | 1.6 (0.2) | 15.6 (8.9-27.6)* | 12.2 (2.6)* | 10.7 (5.0-22.8)* | 8.3 (3.1)* | 24.7 (12.3-49.9)* | 18.7 (4.9)* |
| panic attacks | 199 | 5.5 (0.4) | 6.7 (4.5-9.9)* | 20.5 (3.6)* | 4.9 (2.9-8.4)* | 14.9 (4.2)* | 9.9 (5.7-17.1)* | 29.1 (6.6)* |
| anxiety | 1052 | 29.3 (0.8) | 4.0 (2.9-5.5)* | 44.6 (5.5)* | 3.3 (2.2-4.9)* | 37.9 (7.2)* | 5.3 (3.1-8.9)* | 54.1 (8.8)* |
| alcohol use problems | 37 | 1.0 (0.2) | 6.0 (2.7-13.5)* | 4.0 (1.6)* | 4.9 (1.6-14.5)* | 3.1 (1.7)* | 8.7 (3.1-24.3)* | 6.1 (3.1)* |
| drug use problems (illicit drugs and/or medication) | 50 | 1.6 (0.2) | 6.3 (3.3-12.2)* | 5.6 (1.8)* | 5.7 (2.4-13.3)* | 5.0 (2.2)* | 7.8 (3.2-19.1)* | 7.1 (3.3)* |
| other | 30 | 0.8 (0.2) | 4.0 (1.5-10.9)* | 2.1 (1.2)* | 3.3 (0.9-12.9) | 1.6 (1.4) | 6.1 (1.7-21.4)* | 3.6 (2.1) |
| Number of disorders |  |  |  |  |  |  |  |  |
| two or more | 488 | 13.5 (0.6) | 10.5 (7.1-15.5)* | 43.7 (4.5)* | 7.8 (4.7-13.0)* | 33.0 (5.5)* | 14.9 (8.3-27.0)* | 58.9 (7.1)* |
| exactly one | 738 | 20.9 (0.7) | 3.1 (2.0-4.9)* | 16.9 (4.0)* | 4.0 (2.4-6.7)* | 25.0 (5.5)* | 1.7 (0.7-4.0) | 5.1 (4.9) |
| none | 2274 | 65.7 (0.8) | (ref) | (ref) | (ref) | (ref) | (ref) | (ref) |

a. number of observations (n) are unweighted; proportions (%, SE) are weighted.

b. bivariate models were used, i.e., a separate logistic regression model was created for each type of pre-pandemic lifetime mental disorder and for the number of pre-pandemic lifetime mental disorders.

Abbreviations: OR = odds ratio; CI = confidence interval; IQR = interquartile range; Med = median; PARP = Population Attributable Risk Proportion; SE = standard error; STB = suicidal thoughts and behaviors.

* indicate statistically significant results (α = 0.05); for PARP, statistical significance is based on the percentile bootstrap confidence interval.

**Supplementary Table 6. Associations of proximal risk factors with thirty-day STB (unadjusted analyses; n = 3,500).**

|  |  |  | **Any STB (n = 160)** | | **Passive ideation only (n = 96)** | | **Active suicidal ideation, plan or attempt (n = 64)** | |
| --- | --- | --- | --- | --- | --- | --- | --- | --- |
|  | **n^a^** | **% (SE) or Med (SE) (IQR)^a^** | **OR (95% CI)^b^** | **PARP % (SE)^b^** | **OR (95% CI)^b^** | **PARP % (SE)^b^** | **OR (95% CI)^b^** | **PARP % (SE)^b^** |
| **Positive screens for current mental disorders** |  |  |  |  |  |  |  |  |
| Major Depressive Disorder | 407 | 11.2 (0.5) | 11.0 (7.9-15.4)* | 47.8 (4.5)* | 7.2 (4.7-11.0)* | 35.8 (6.0)* | 21.3 (12.4-36.5)* | 65.3 (6.7)* |
| Generalized Anxiety Disorder | 395 | 10.9 (0.5) | 10.2 (7.3-14.2)* | 45.1 (4.3)* | 6.8 (4.5-10.4)* | 33.8 (5.8)* | 18.8 (11.1-31.8)* | 61.6 (6.8)* |
| Post-traumatic Stress Disorder | 342 | 9.5 (0.5) | 7.3 (5.2-10.2)* | 33.2 (4.2)* | 6.7 (4.3-10.3)* | 31.1 (5.3)* | 8.2 (4.9-13.7)* | 36.6 (6.9)* |
| Panic Attacks | 357 | 9.8 (0.5) | 11.1 (7.9-15.5)* | 44.3 (4.4)* | 8.3 (5.4-12.7)* | 36.2 (5.6)* | 17.1 (10.3-28.6)* | 56.2 (6.9)* |
| Alcohol or Substance use Disorder | 94 | 2.8 (0.3) | 5.6 (3.3-9.5)* | 9.0 (2.5)* | 3.5 (1.6-7.6)* | 4.9 (2.5)* | 9.5 (4.9-18.6)* | 15.8 (5.0)* |
| Number of positive screens |  |  |  |  |  |  |  |  |
| two or more | 426 | 11.8 (0.6) | 23.8 (15.7-36.2)* | 55.1 (4.0)* | 14.7 (8.9-24.3)* | 43.6 (5.5)* | 55.4 (24.9-123.5)* | 71.4 (6.1)* |
| exactly one | 418 | 11.8 (0.6) | 7.9 (4.9-13.0)* | 19.3 (3.6)* | 7.1 (4.1-12.5)* | 22.6 (5.1)* | 10.8 (4.1-28.1)* | 14.8 (4.9)* |
| none | 2656 | 76.4 (0.7) | (ref) | (ref) | (ref) | (ref) | (ref) | (ref) |
| **Personal Health** |  |  |  |  |  |  |  |  |
| History of COVID-19 infection or isolation/quarantine for COVID-19 | 600 | 16.5 (0.6) | 0.7 (0.4-1.1) | -5.6 (2.8) | 0.6 (0.3-1.1) | -6.6 (3.5) | 0.8 (0.4-1.7) | -1.6 (4.5) |
| Number of close contacts (<1 meter) when working outside of home |  | 0.0 (0.2) (0.0-0.0) | 1.0 (1.0-1.0) | -1.5 (1.8) | 1.0 (1.0-1.0) | 0.1 (2.2) | 1.0 (1.0-1.0) | -2.9 (4.4) |
| Perceived inefficiency of protective equipment at work (scaled 0-4) |  | 0.0 (0.1) (0.0-0.0) | 1.4 (1.1-1.7)* | 4.8 (2.3)* | 1.4 (1.1-1.8)* | 5.1 (2.8)* | 1.4 (1.0-1.9) | 5.0 (4.1) |
| Stress about personal health (scaled 0-4) |  | 0.7 (0.0) (0.0-1.6) | 1.7 (1.5-1.9)* | 50.0 (6.7)* | 1.5 (1.2-1.8)* | 37.0 (9.4)* | 2.1 (1.7-2.6)* | 64.9 (8.3)* |
| **Health of loved ones** |  |  |  |  |  |  |  |  |
| Having loved ones infected with COVID-19 | 1606 | 45.0 (0.9) | 1.4 (1.1-2.0)* | 16.4 (7.2)* | 1.5 (1.0-2.2) | 16.9 (9.6) | 1.4 (0.9-2.3) | 16.2 (11.6) |
| Stress about health loved ones (scaled 0-4) |  | 1.7 (0.0) (0.8-2.6) | 1.4 (1.2-1.6)* | 53.5 (7.8)* | 1.4 (1.2-1.7)* | 49.9 (10.9)* | 1.5 (1.2-1.9)* | 57.6 (11.0)* |
| **Financial factors** |  |  |  |  |  |  |  |  |
| Significant income loss or (temporarily) unemployed due to COVID-19 | 1445 | 39.8 (0.8) | 1.2 (0.8-1.6) | 5.7 (6.8) | 1.1 (0.7-1.7) | 4.4 (8.7) | 1.2 (0.7-2.0) | 7.7 (10.7) |
| Stress about financial situation (scaled 0-4) |  | 0.9 (0.0) (0.0-2.1) | 1.2 (1.1-1.4)* | 25.6 (7.9)* | 1.2 (1.0-1.4) | 18.4 (10.8) | 1.3 (1.1-1.6)* | 35.6 (11.5)* |
| **Interpersonal factors** |  |  |  |  |  |  |  |  |
| Interpersonal stress (scaled 0-4) |  | 0.6 (0.0) (0.0-1.4) | 1.7 (1.5-2.0)* | 45.7 (5.3)* | 1.5 (1.2-1.9)* | 35.2 (7.7)* | 2.1 (1.6-2.6)* | 58.5 (6.4)* |
| Lack of social support (scaled 0-4) |  | 0.8 (0.0) (0.4-1.3) | 2.0 (1.6-2.4)* | 54.8 (7.5)* | 1.5 (1.1-1.9)* | 31.5 (14.0)* | 2.9 (2.2-3.9)* | 75.0 (7.1)* |

a. number of observations (n) are unweighted; proportions (%, SE) and medians (Med, IQR) are weighted.

b. bivariate models were used, i.e., a separate logistic regression model was created for each proximal risk factor.

Abbreviations: OR = odds ratio; CI = confidence interval; IQR = interquartile range; Med = median; PARP = Population Attributable Risk Proportion; SE = standard error; STB = suicidal thoughts and behaviors.

* indicate statistically significant results (α = 0.05); for PARP, statistical significance is based on the percentile bootstrap confidence interval.

**Supplementary Table 7. Associations of having loved ones infected by COVID-19 with thirty-day STB (n = 3,500).**

|  |  |  | **Any STB (n = 160)** | **Passive ideation only (n = 96)** | **Active suicidal ideation, plan or attempt (n = 64)** |
| --- | --- | --- | --- | --- | --- |
|  | **n^a^** | **% (SE)^a^** | **OR (95% CI)^b^** | **OR (95% CI)^b^** | **OR (95% CI)^b^** |
| **Type of loved ones infected with COVID-19** |  |  |  |  |  |
| partner, children, or parents | 197 | 5.4 (0.4) | 2.2 (1.1-4.3)* | 1.4 (0.5-3.6) | 4.2 (1.8-10.0)* |
| other family, friends or others (but no partner, children, or parents) | 1409 | 39.6 (0.8) | 1.6 (1.1-2.3)* | 1.7 (1.1-2.6)* | 1.4 (0.8-2.4) |
| none of the above | 1894 | 55.0 (0.9) | (ref) | (ref) | (ref) |
| **Severity COVID-19 infection of most affected loved one** |  |  |  |  |  |
| died | 303 | 8.7 (0.5) | 1.3 (0.7-2.3) | 1.5 (0.8-3.0) | 1.1 (0.4-2.8) |
| hospitalized | 473 | 13.1 (0.6) | 1.9 (1.2-3.0)* | 1.7 (1.0-3.1) | 2.0 (1.0-4.0) |
| severe symptoms | 300 | 8.2 (0.5) | 2.1 (1.2-3.6)* | 1.4 (0.7-3.1) | 3.1 (1.5-6.4)* |
| light symptoms | 450 | 12.7 (0.6) | 1.4 (0.8-2.4) | 1.6 (0.9-3.0) | 1.0 (0.4-2.4) |
| no symptoms | 1974 | 57.3 (0.9) | (ref) | (ref) | (ref) |

a. number of observations (n) are unweighted; proportions (%, SE) are weighted.

b. adjusted models were used, i.e., a separate logistic regression model was created for type of loved ones infected and for severity of infection, each time adjusting for distal risk factors.

Abbreviations: OR = odds ratio; CI = confidence interval; IQR = interquartile range; Med = median; SE = standard error; STB = suicidal thoughts and behaviors.

* indicate statistically significant results (α = 0.05).
